# Electronic structure of two-dimensional In and Bi metal on BN nanosheets

**DOI:** 10.1039/c9ra00673g

**Published:** 2019-03-22

**Authors:** Maolin Bo, Jibiao Li, Chuang Yao, Zhongkai Huang, Lei Li, Chang Q. Sun, Cheng Peng

**Affiliations:** Key Laboratory of Extraordinary Bond Engineering and Advanced Materials Technology (EBEAM) of Chongqing, Yangtze Normal University Chongqing 408100 China leili16@yznu.edu.cn 20090008@yznu.cn; NOVITAS, School of Electrical and Electronic Engineering, Nanyang Technological University Singapore 639798 Singapore

## Abstract

The electronic structures of two-dimensional (2D) indium (In) and bismuth (Bi) metal on BN nanosheets are systematically studied using hybrid density functional theory (DFT). We found that 2D In and Bi metal effectively modulate the band gap of a BN nanosheet. We also found that the indirect band gap of the 2D In and Bi metal electronic structures are 0.70 and 0.09 eV, respectively. This modulation originates from the charge transfer between the 2D metal and BN nanosheet interfaces, as well as from the electron redistribution of the In/BN and Bi/BN heterojunctions of the s and p orbitals. Our results provide an insight into 2D In/BN and Bi/BN heterojunctions, which should be useful in the design of 2D In and Bi metal–semiconductor-based devices.

## Abstract 2

The electronic structures of two-dimensional (2D) indium (In) and bismuth (Bi) metal on BN nanosheets are systematically studied using hybrid density functional theory (DFT).

## Graphical abstract

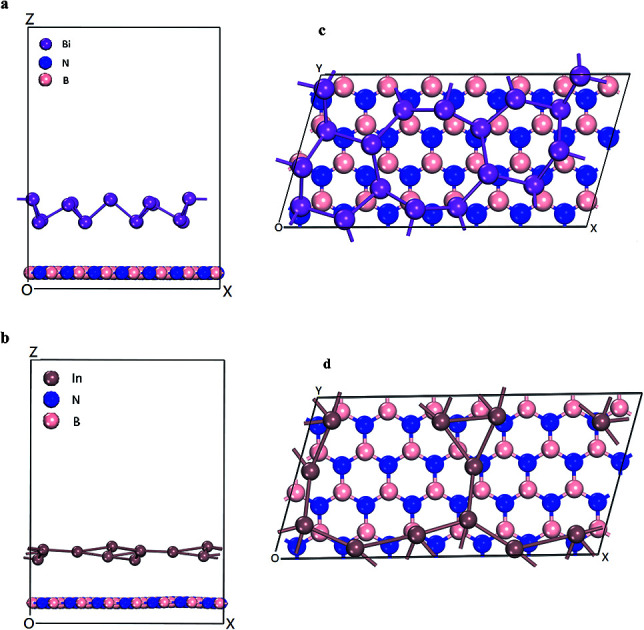

## 1. Introduction

Typical two-dimensional (2D) materials include graphane, MoSe_2_, WS_2_, MoS_2_, WSe_2_, and black phosphorus.^1–4^ These 2D semiconductor materials have excellent optical and electronic properties, as well as high surface area to volume ratios and a low coordination of their surface atoms.^5^ This low coordination of the surface atoms causes the surface atomic bonds to contract, which in turn influences the electronic densification, polarization, charge transfer and local bond strains.^6^ Therefore, reducing the number of atomic layers can regulate the band gap, as well as the electronic, electrochemical, optical, and catalytic properties.

Recently, researchers have reported on monoatomic layers of As, Bi, and Sb metal.^7,8^ Zhang *et al.* found that single atomic layers of As and Sb metal form indirect semiconductors with band gaps of 2.49 and 2.28 eV, respectively.^9^ Reis *et al.* undertook a combined theoretical and experimental study of monolayer Bi metal grown on a SiC(0001) substrate to achieve an indirect gap of 0.67 eV.^10^ The conversion of metallic conductors to semiconductors through a reduction in the number of atomic layers,^11^ and the resulting electrochemical, optical, and catalytic and electronic properties, is an important aspect of these materials that warrants careful study.

In an actual 2D device, the 2D materials typically have to be in contact with a metal or semiconductor substrate.^7,12,13^ Shi *et al.* synthesized large-scale and high-quality Sb metal on a WTe_2_ substrate.^14^ Simultaneously, when 2D materials are stacked together, they can develop surprising electronic and photocatalytic properties.^15,16^ For example, Wang *et al.* reported that a SrTiO_3_/NaTaO_3_ heterojunction exhibits improved SrTiO_3_ photocatalytic properties.^17^ Zhang *et al.* found that MoS_2_/Ni_3_S_2_ heterostructures have highly electrochemical properties.^18^ Rivera *et al.* observed interlayer excitons in a MoSe_2_/WSe_2_ heterostructure when subjected to photoluminescence excitation spectroscopy.^19^ Therefore, two-dimensional heterojunctions can change the electronic states, band gap, and photocatalytic properties, as well as the physical and chemical properties that can be used in the design of new devices.^20–22^

In the present study, we determined the band gap of a single honeycomb BN sheet to be 4.76 eV. Density functional calculations showed that the band gap can be tuned using Bi/BN and In/BN heterostructures, for which the band gaps are 0.70 and 0.16 eV, respectively. Therefore, we can change the band structure by adding 2D metal to a 2D semiconductor. The results of the present study provide a new route to the design of desirable semiconductors using 2D metal and semiconductor heterostructures.

## 2. Methods

**Fig. 1:**
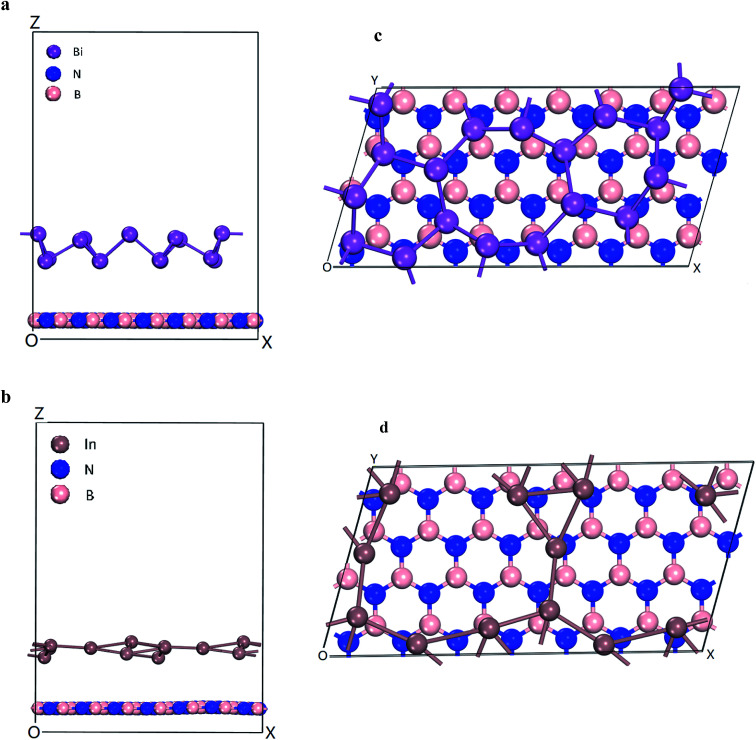
Heterostructures of Bi/BN and In/BN (a and b) side views and (c and d) top views.

The energetics and electronic properties of Bi, In, BN, Bi/BN, and In/BN structures were analyzed from first principles. The DFT calculations used the projector-augmented wave potentials of the hybrid density functional, which was implemented in the CASTEP software. We used the HSE06 (ref. 23) hybrid density function to describe the electron exchange and correlation potential. The 2D stable structures of Bi, In, BN, Bi/BN, and In/BN are shown in Fig. 1. For the DFT calculations, we used the Vienna ab initio simulation package (VASP) to optimize the initial structure such that the structure would converge faster. Then, we used the CASTEP software to optimize the structure and other properties. We assumed that the Bi/BN and In/BN heterostructures of the atomic layer would influence the van der Waals force and relax the lattice constant and atomic position. The plane wave cut-off value was 700 eV. The *k*-points of the Brillouin zone were employed for sampling to relax the lattice constants of 2 × 4 × 1. The vacuum space was approximately 16 Å. In the calculations, the criteria affecting the atom force convergence and energy were set to 10^−6^ eV and 0.01 eV Å^−1^, respectively.

## 3. Results and discussion

We calculated the formation energy of the Bi/BN and In/BN heterostructures. The formation energy *E*_form_ is defined as:*E*_form_ = *E*^heterostructure^_total_ − *E*^Bi(In)^_total_ − *E*^BN^_total_ (eV)

These negative formation energies mean that the 2D heterostructures are energetically favorable.^24^ A lower *E*_form_ value means that the calculated heterostructures have a lower energy state and a more stable configuration, suggesting that the structures could be synthesized in the laboratory. The formation energies *E*_form_ are shown in Table 1.

**Table 1:** Heterojunction formation energy

*E* ^heterostructure^_total_ (eV)		*E* ^In(Bi)^_total_ (eV)		*E* ^BN^_total_ (eV)		*E*_form_ (eV)
In/BN	−10 431.09	In	−647.19	BN	−9779.61	−4.29
Bi/BN	−43 134.74	Bi	−33 349.13	BN	−9779.60	−6.01

The electronic structures and band gaps of Bi, In, BN, Bi/BN, and In/BN were calculated. For the Bi/BN and In/BN heterostructures, we considered the influence of the van der Waals forces, assuming the changes in the internal plane structure to be small and negligible. As can be seen from Fig. 1, the two-dimensional structure requires some spacing to maintain its stability. We set the initial vertical distance between the bottom BN layer and the top metallic layer to 3.0 Å. The atomic layer distances between the two monolayers in the heterostructures, after relaxation, are listed in Table 2. The layer distances of the Bi/BN and In/BN heterostructures are 4.45 and 3.77 Å, respectively.

**Table 2:** Correspondence between settings of atomic layer spacing and band gaps of Bi/BN and In/BN heterojunctions^a^

Structures	Layer spacing (Å)	Band gap (eV)	Angles			Lattice parameters		
			*α*	*β*	*γ*	*a*	*b*	*c*
Bi/BN	4.45	0.70	88.54°	88.62°	73.99°	17.59 Å	9.04 Å	23.05 Å
In/BN	3.77	0.16	89.91°	89.98°	73.97°	17.58 Å	9.06 Å	23.00 Å

a Angles and lattice parameters of Bi/BN and In/BN heterojunctions.

In the Bi/BN and In/BN heterostructures, the average bond lengths of the In–In and Bi–Bi metal bonds are 3.107 and 3.067 Å, respectively. The In–In and Bi–Bi metal bulk bond lengths are 4.36 and 3.379 Å, respectively. The In–In and Bi–Bi bond lengths in the Bi/BN and In/BN heterostructures were found to be slightly shorter than the In and Bi metal bulk bond lengths, which can be attributed to the reconstruction of the surface atoms to accommodate the surface bond contraction caused by the lattice strain. As shown in Fig. 1, the metals have a plane geometric structure. Meanwhile, the Bi metal has a curved honeycomb geometric structure. The In–In metal and Bi–Bi metal bond angles in the Bi/BN and In/BN heterostructures are 78.54–99.19° and 106.16–166.82°, respectively. This curved honeycomb structure of the Bi metal helps to stabilize the layered structure, in the same way as blue phosphorene, germanene, silicene, and stanene.

**Fig. 2:**
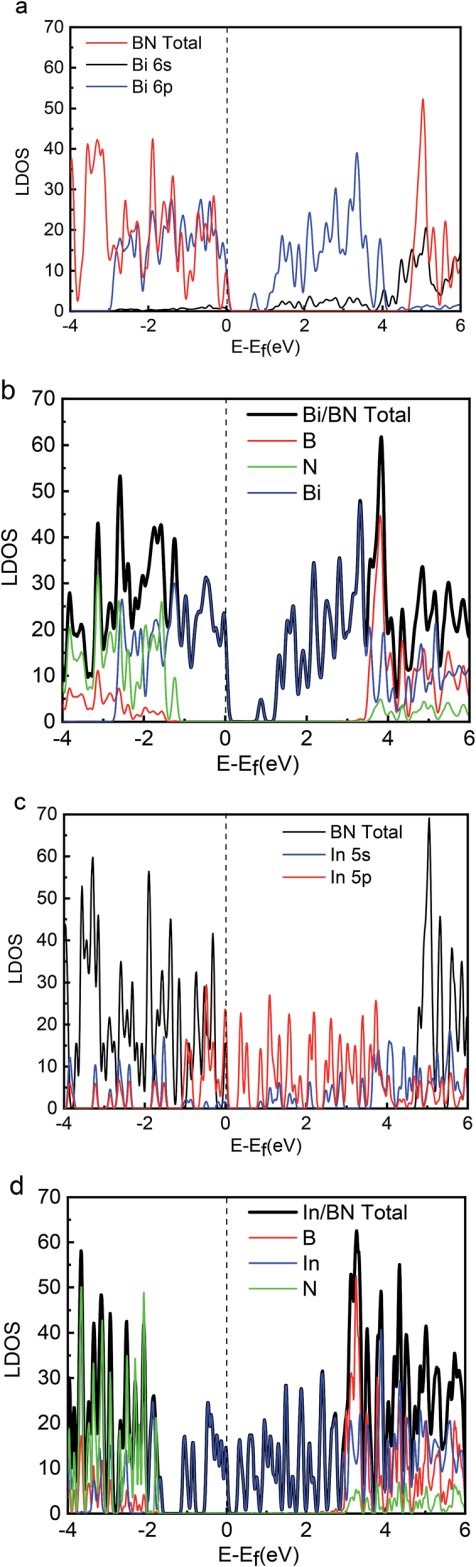
LDOS values for Bi, In, BN, Bi/BN, and In/BN structures.

Energy distribution is also important to physical and chemical systems. The energy distribution determines the state of the system and the electronic properties of the material. The band gap, valence band, and conduction band can be clearly obtained from the DOS diagram. The local densities of states (LDOS) for the 2D structures of Bi, In, BN, Bi/BN, and In/BN are shown in Fig. 2. For the Bi and In metals, the conduction-band bottom and valence-band top mainly consists of Bi 6p^3^ (In 5p^1^) states coupled with small amounts of Bi 6s^2^ (In 5s^2^) states. The semiconductor characteristics of the monolayer Bi and In metal are mainly due to the atomic p and s orbitals at the Fermi level.

**Fig. 3:**
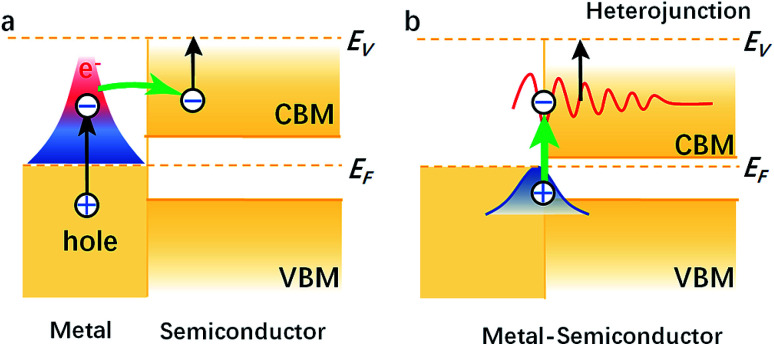
VBM and CBM of (a) metal, semiconductor and metal (b) metal–semiconductor heterostructures.

We compared the LDOS values of the Bi 6s orbit, Bi 6p orbit, and BN, as shown in Fig. 2(a). Fig. 2(b) shows the LDOS values of the Bi/BN heterostructures. Differences can be seen in the DOS values of the BN sheet and Bi/BN heterostructures at the Fermi level. The electronic contribution of the BN sheet to the DOS can be attributed to the B and N atoms at the Fermi level. However, the electronic contribution of the DOS of the Bi/BN heterostructures at the Fermi level is mainly caused by the Bi metal. Similar DOS values were also observed for the BN and Bi/BN heterostructures, as shown in Fig. 2(c) and (d). The s and p electrons of In and Bi metal fill the top of the valence band of the BN sheets. Fig. 3 shows that the electronic contribution and charge transfer of the valence band maximum (VBM) and conduction band minimum (CBM) of the Bi/BN and In/BN heterostructures at the Fermi level are mainly caused by the Bi and In metal. These results are in good agreement with the latest model of electron transfer at a Ag/graphite heterojunction.^25^

Our computations show that monolayer Bi and In metal feature a semiconductor band gap. To gain further insights, the VBM and the CBM of Bi and In are mainly composed of the p and s orbitals of electrons. We found that the main peak of the DOS in the CBM lies at 0.70 eV for Bi, 0.09 eV for In, 4.76 eV for BN, 0.16 eV for Bi/BN, and 0.70 eV for In/BN. Most importantly, we observed that the band gap of the metal Bi is 0.70 eV. These results demonstrate that a new band gap has been formed in the metallic Bi. Therefore, the two-dimensional structure of the Bi metal has been converted into a semiconductor material. Similar electronic structure transitions from a metal to a semiconductor are also observed for the monolayer of metallic In.

**Fig. 4:**
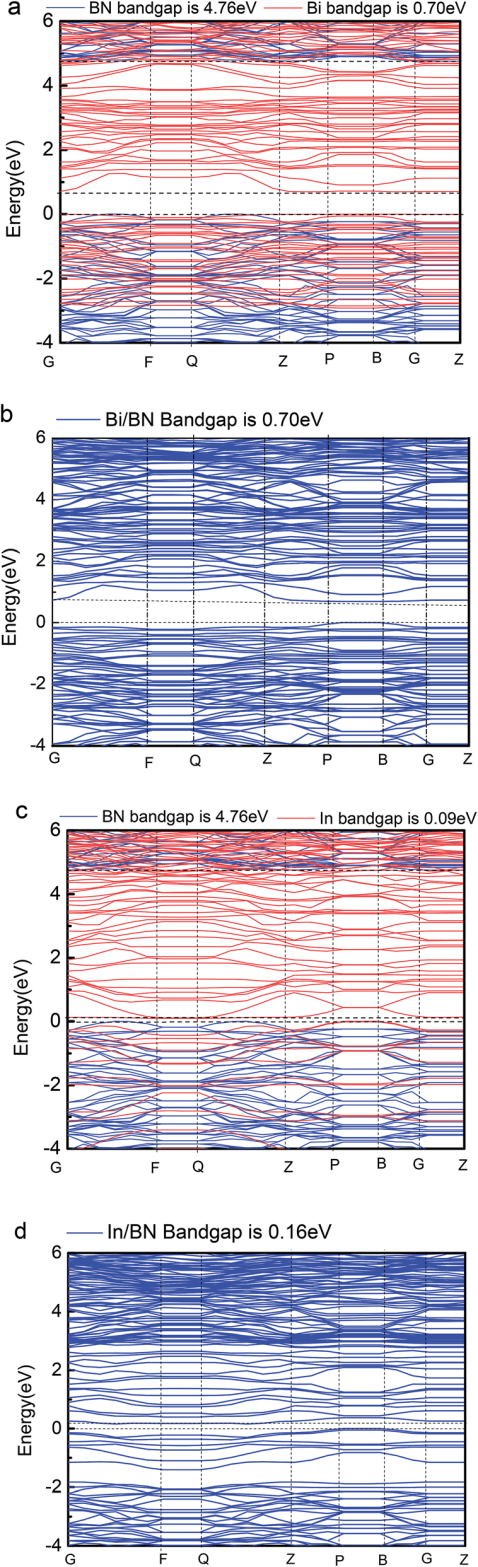
Band gaps of Bi, In, BN, Bi/BN, and In/BN structures.

Geometric structure determines the electronic properties. The Fig. 4(b), Bi/BN band gap is 0.7 eV which is same to the band gap of Bi (0.7 eV) in Fig. 4(a). The geometric structure of Bi metal is comparable to the geometric structure of Bi on BN sheet; we found that its atomic position does not change much. The In/BN band gap is 0.16 eV which is larger than the band gap of In (0.09 eV) in Fig. 4(c). We found some changes in the atomic positions, as shown in the Table 3. Fig. 4 shows not only the band gaps of the Bi/BN and In/BN heterostructures, but also those of the monolayers of Bi, In, and BN. The band gaps of the Bi/BN and In/BN heterostructures, as calculated by hybrid DFT, are 0.70 and 0.16 eV, respectively, which are 4.06 and 4.60 eV lower than the corresponding values for the monolayer BN sheet. Previous experimental and theoretical studies of the band gaps of Bi on a SiC substrate yielded a value of 0.67 eV (ref. 10) while for a single honeycomb BN sheet, the value was 4.80 eV.^26^ We studied the Bi/BN and In/BN heterostructures, finding that the band gap is smaller than that of the BN sheet, implying that the band structure can be adjusted by using 2D metal with semiconductor properties.

**Table 3:** Atomic positions of Bi and In metal

	*X*	*Y*	*Z*	*X*	*Y*	*Z*
	Bi/BN			Bi		
Bi	2.71849	8.04324	7.88434	2.69243	8.02398	7.82213
Bi	3.85548	0.60316	7.37952	3.86017	0.63672	7.40532
Bi	9.55824	6.84850	7.83619	9.55131	6.89164	7.74978
Bi	11.60958	5.72985	5.88509	11.63010	5.71193	5.91919
Bi	16.13413	6.79861	5.91527	16.07899	6.78490	5.96873
Bi	2.83437	5.61181	6.01296	2.88017	5.57781	6.04338
Bi	1.34126	1.30908	5.82265	1.38841	1.30555	5.87702
Bi	5.88252	2.31468	5.84223	5.84379	2.34036	5.89016
Bi	7.87431	1.19976	7.85439	7.87135	1.16233	7.76660
Bi	13.60277	7.44120	7.46776	13.57875	7.42737	7.46684
Bi	17.28458	8.75694	7.95300	17.31905	8.73475	7.90554
Bi	14.63224	2.50331	6.09407	14.61604	2.50333	6.14430
Bi	5.40258	4.87267	7.51546	5.42228	4.89841	7.50947
Bi	7.21669	6.78895	5.86159	7.16932	6.84462	5.89615
Bi	10.25366	1.24239	5.92633	10.29354	1.22881	5.96412
Bi	12.04020	3.18445	7.58550	12.04791	3.18310	7.57306
Bi	16.07444	4.50836	7.93710	16.09822	4.49188	7.90992
Bi	1.44893	3.58079	7.86522	1.42300	3.59027	7.82697
	In/BN			In		
In	4.41351	0.54878	6.24623	4.73354	0.69648	6.17675
In	13.04933	0.38548	6.25520	13.50201	0.68650	6.19401
In	2.04916	4.74593	5.54666	1.78949	4.79518	6.14179
In	10.90435	4.87502	5.53906	10.65058	4.86892	6.14754
In	9.16040	7.34008	5.82363	9.03537	7.23983	6.17652
In	17.82898	7.19784	5.88436	17.74978	7.17751	6.18043
In	7.75626	1.22207	6.70586	7.73500	1.14958	6.16801
In	16.40263	1.13095	6.72311	16.48652	1.07629	6.17721
In	3.25284	7.49042	6.21517	3.02228	7.48151	6.14018
In	12.19995	7.58643	6.26118	11.87791	7.56562	6.15030
In	1.68308	1.82715	6.42129	1.91794	1.77129	6.15881
In	10.54597	2.00315	6.35550	10.74625	1.84477	6.16617

Layered Bi/BN and Bi/BN heterojunctions configured using Bi and In metal were synthesized in the laboratory using molecular beam epitaxy. We could control the band structure by changing the local atomic orbital interactions. Given the special electronic properties, the deformation charge density can be used to determine the characteristics of the atomic bond through the corresponding energy density distribution. Negative charge density regions cause electrons to diverge and positrons to accumulate, forming charge-density regions. The transfer of the charge is represented by a deformation charge density map using different colors. The charge accumulated between two atoms forms a covalent bond; however, charge transfer or divergence gives rise to an ionic bond. For example, the point at which the covalent bond electrons converge is between two atoms, such that an ionic bond converges to one side.

**Fig. 5:**
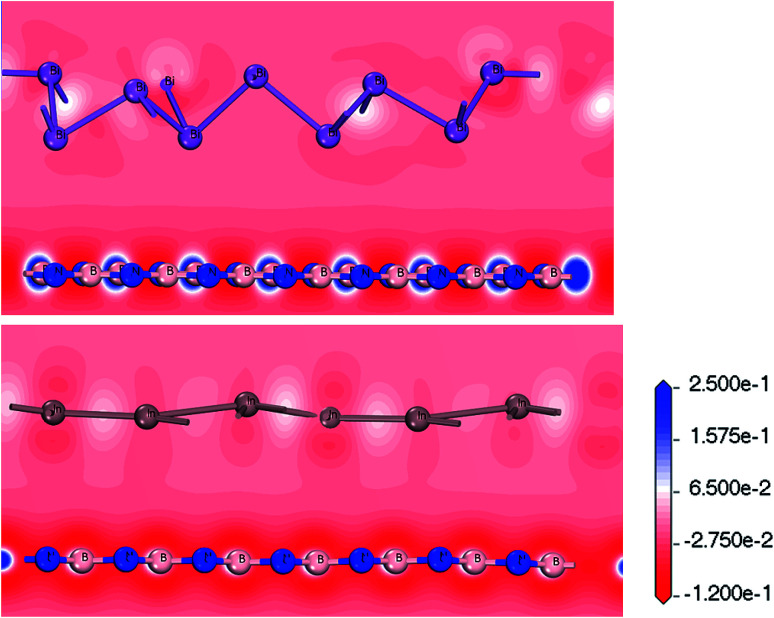
Charge density difference between Bi/BN and In/BN heterostructures.

Fig. 5 shows the deformation charge density. We believe that the formation of the Bi/BN and Bi/BN heterostructures is mainly due to the transfer of the charge and electronic density distribution. The scale indicates the charge distribution. The increase and decrease in the number of electrons is shown in blue and red, respectively. For the Bi/BN and In/BN heterostructures, the BN nanosheet deformation charge density is shown in red and blue. That is to say, a positive deformation charge density causes the electron density to increase. In contrast, the deformation charge density of the 2D In and Bi metals is mainly shown in red. The deformation charge density is negative, indicating that electrons are lost from these parts. We examined the Bi/BN and Bi/BN heterostructures and found that they all have indirect band gaps and interlayer coupling.

## 4. Conclusion

We studied the electronic properties and atomic bonding of Bi/BN and In/BN heterojunctions containing 2D In and Bi metals, and compared the results with the corresponding monolayer 2D metals and BN nanosheets. The band gap of In/BN and In are 0.16 eV and 0.09 eV, respectively. The Bi/BN band gap is 0.7 eV which is same to the band gap of Bi. The calculated band gaps for Bi, In, BN, Bi/BN, and In/BN indicate that these 2D metal and metal–semiconductor heterojunctions have suitable band gaps. Theoretical predictions indicate that Bi, In, BN, Bi/BN, and In/BN are promising candidates for new 2D materials. These findings will be useful to the design of related optoelectronic devices.

## Conflicts of interest

The authors declared that they have no conflicts of interest to this work.

## Supplementary Material
